# User acceptability profiles of vaginal treatments among women with genitourinary syndrome of menopause

**DOI:** 10.1371/journal.pone.0331110

**Published:** 2025-09-29

**Authors:** Mohammed Hassanein, Hasniza Zaman Huri, Abduelmula Abduelkarem

**Affiliations:** 1 Department of Clinical Pharmacy and Pharmacy Practice, Faculty of Pharmacy, Universiti Malaya, Kuala Lumpur, Malaysia; 2 Department of Pharmacy Practice and Pharmacotherapeutics, College of Pharmacy, University of Sharjah, University City Road, University City, Sharjah, United Arab Emirates; 3 Research Institute for Medical and Health Sciences, University of Sharjah, University City Road, University City, Sharjah, United Arab Emirates; Independent Consultant, UNITED STATES OF AMERICA

## Abstract

**Background:**

This study aimed to identify patient segments of acceptability to vaginal dosage forms in women with the genitourinary syndrome of menopause and to identify the most important predictors and mediators of each segment using latent segment analysis.

**Methods:**

A cross-sectional study included 351 peri- and postmenopausal women from two tertiary care hospitals in the United Arab Emirates. To select the best model, we ran a finite-mixture partial least squares segmentation (FIMIX-PLS). The number of resulting segments was used to run a partial least squares-predicted-oriented segmentation to assign cases to segments and maximize the segment-specific explained variance (R^2^) across all groups. Multi-group analysis was then performed to examine whether differences between segments were significant. Analyses were performed using the SmartPLS Software version 4.9,

**Results:**

A two-segment model was identified (Entropy > 0.8, Corrected-Akaike’s Information Criterion = 1977.11; Bayesian Information Criterion = 1960.11), which indicated adequate and well-separated segments. The first and second segments had 179 and 171 of the cases, respectively, which were considered substantive representations of both segments. The model resulted in a weighted average R^2^ greater than that of the original sample, indicating better predictive relevance of the model. The multi-group analysis showed that the differences between the two segments were significant. Being on vaginal treatment was observed to be the variable that drove the partition of the segments. Perceived effectiveness for those who were not on treatment (segment 1) and those who were (segment 2) was predicted by affective attitude and intervention coherence, respectively,

**Conclusion:**

The acceptability of vaginal treatments differs between experienced and anticipated users. Considering patient-related factors and previous treatment experiences can serve as a benchmark to improve patient acceptability of treatment.

## Introduction

Women with the genitourinary syndrome of menopause (GSM) frequently experience vaginal, urinary, and sexual symptoms. Current clinical guidelines including the 2020 position statement of the North American Menopause Society and the British Menopause Society, recommend local vaginal treatments as the preferred route if the only complaint is urogenital symptoms rather than vasomotor symptoms, which usually require systematic therapy [1–2]. Despite these recommendations, a significant and often neglected barrier to the widespread use of vaginal preparations is women’s acceptance and willingness to utilize them. Because vaginal products for GSM are intended for long-term therapeutic use rather than preventive use, women may be more selective about their attributes to ensure effective relief and comfortable use.

Many women do not seek treatment due to embarrassment, lack of awareness, or the belief that symptoms are a normal part of aging [3–4]. A Woman’s preference regarding using a vaginal treatment can be influenced by several factors including the characteristics of the product itself, the method of administration, cultural factors, and the woman’s past experiences [5]. On the other hand, factors like perceived effectiveness, intervention coherence, self-efficacy, and affective attitude toward the treatment are crucial aspects of the user experience that had not been widely investigated. Data from large-scale surveys including the REVIVE (Real Women’s views of Treatment Options for Menopausal Vaginal Changes), the VIVA (Vaginal health: Insights, Views & Attitudes), and the EMPOWER surveys, indicate that current treatment users demonstrate no significant preference between orally and vaginally administered therapies, with both routes yielding relatively comparable overall treatment satisfaction [6–8]. However, among treatment-naïve patients, a distinct preference for oral therapeutic options was observed [9].

Treatment preferences and acceptability represent two relevant but distinct concepts. Treatment preference was defined to represent patient’s choice of treatment to manage their clinical condition among other alternative treatments. Acceptability, on the other hand, was defined as the positive or favourable attitude toward these treatment options [10]. Therefore, treatment preference stems from the patient’s acceptability of the treatment. Later, Sekhon et al. developed the Theoretical Framework of Acceptability (TFA) that suggested that treatment acceptability is a multidimensional concept that is represented by seven constructs. [11–12]. It reflects how appropriate the intervention is considered by either the patients or health care providers (HCPs) based on their cognitive and emotional responses [11]. Understanding and measuring treatment acceptability can significantly impact the success and adherence to various interventions. The practical efficacy of a treatment is dependent on the acceptability to effectively use it. Specifically, it is important to consider variations in perceptions of acceptability and preferences among different age groups as well as individuals with different conditions or intended uses [5]. This study aimed to identify distinct users profiles regarding the acceptability of vaginal dosage forms and to investigate the driving factors that contribute to a GSM patient’s fitting to a particular profile.

## Methods

### Study settings and participants

A cross-sectional observational study was conducted on 351 participants who were purposefully sampled from two distinct tertiary hospitals in the United Arab Emirates between 5^th^ June 2023 and 5^th^ November 2023. The target population included peri- and postmenopausal women defined according to the STRAW guidelines [13] who were diagnosed with urogenital atrophy or who presented with urogenital symptoms. Because acceptability can be assessed retrospectively and prospectively, women who were currently receiving treatment and those who had already completed treatment or were expected to use a vaginal treatment for symptom management were considered eligible for inclusion in the study. Participants who were unable to understand the study procedures, risks, and benefits, who could not voluntarily agree to participate or were unable to understand or read the study questionnaire in either Arabic or English were excluded. This study was approved by the Ministry of Health and Prevention Ethics Committee (reference number MOHAP/DXB-REC/OON/No.106/2022) and have been conducted according to the principles expressed in the Declaration of Helsinki. All participants provided informed written consent before the administration of the study questionnaire.

### Study instrument

**Fig 1:**
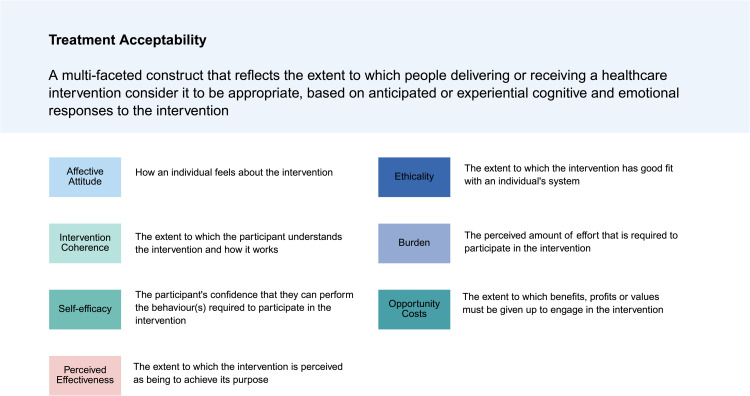
Theoretical Framework of Acceptability.

The genitourinary syndrome of menopause symptoms and vaginal treatment acceptability questionnaire (GSM-SVTAQ) is a GSM-specific, multi-dimensional patient-reported outcomes measure [14]. The GSM-SVTAQ includes a 13-item scale that evaluates the acceptability of vaginal treatments across the seven distinct constructs of the TFA [11]. In our model, the study assessed four key constructs—affective attitude, intervention coherence, self-efficacy, and perceived effectiveness — using a 5-point Likert scale (ranging from strongly disagree to strongly agree). Items of treatment acceptability were based on the constructs of the TFA, and their definitions as shown in Fig 1. [11–12].

Adopted from [11]. The common creative license of this figure source is provided through this link: http://creativecommons.org/publicdomain/zero/1.0/.

### Conceptual framework

**Fig 2:**
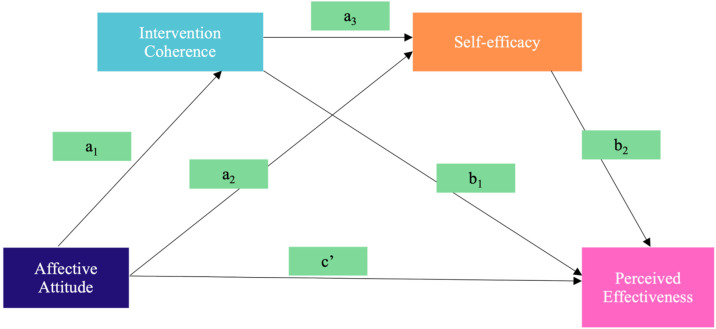
Conceptual Framework.

Our selection of intervention coherence, affective attitude, self-efficacy, and perceived effectiveness was based on their fundamental interconnections within a proposed conceptual framework (Fig 2), which guides our assessment of intervention acceptability. This framework illustrates the relationships between factors influencing how women may perceive the effectiveness of an intervention in which this perception of effectiveness is a primary determinant of an intervention’s overall acceptability. Within this model, intervention coherence and self-efficacy function as both exogenous and endogenous variables. Perceived effectiveness, as the model’s endogenous variable, represents the ultimate outcome of these interplays. The framework hypothesized several pathways where intervention coherence can directly impact perceived effectiveness or indirectly, through the mediating effect of self-efficacy. Affective attitude may directly influence perceived effectiveness or indirectly through the mediating effects of either intervention coherence, self-efficacy, or both in a parallel mediating model.

### Study procedures

Eligible patients were invited by the site sub-investigator for a brief discussion on the nature of the study, its objectives, and the nature of participation. These discussions were primarily after the patient’s visit with her gynecologist was over to ensure her comfort. The participant was prompted to a link that gives her access to the participant information sheet. Participants were then encouraged to provide their consent before they could have access to the study questionnaire. If the patient showed a willingness to participate, she was then given access to the structured data collection form and the GSM-SVTAQ through a scanned QR code, which was placed at a convenient location at the clinic. The structured form was developed to collect data on the socio-demographic of participants. All study materials were provided in English and Arabic so that participants choose the language of their preference.

### Statistical methods

To identify unobserved (hidden) subgroups of acceptability to vaginal treatments among menopausal women, a person-centred approach was required to define the heterogeneous subgroups that can potentially benefit from a common intervention, emphasizing that we did not have an explanatory variable that identifies these groups in meaningful clinical profiles. Latent segment analysis aims to detect unobserved heterogeneity within a sample based on how participants answer a variety of questions about the treatment (observed variables). This “person-centered” approach helps describe *who* these women are, rather than just how individual factors relate to each other [15–17]. Unlike variable-centered approaches, person-centered approaches are meant to describe the subject herself accurately and adequately.

To determine the optimal number of latent segments and their respective sizes, a finite mixture segmentation-partial least squares (FIMIX-PLS) was performed. The FIMIX-PLS was first introduced by Hann et al. [18] and expanded upon by Sarstedt et al. [19]. The advantage of applying the parametric finite mixture regression concept to partial least squares (PLS) path models is that it offers segment retention criteria for model selection [19]. Moreover, unlike traditional clustering techniques that ignore the path relationships that are likely to be responsible for part of the differences between groups, FIMIX-PLS considers this path relationship and looks for unobserved heterogeneity in the structural model; it helps select the best model by looking at how well these relationships explain the differences between the groups [20]. Although FIMIX-PLS is great for finding the initial groups and understanding their internal relationships [21], it does not account for the heterogeneity in the measurement model. [22]. Therefore, to maximize the explained variances across segments and increase the predictive relevance of the model, we used the defined number of segments resulting from the FIMIX-PLS to run a partial least squares-prediction oriented segmentation (PLS-POS). It uses a PLS-specific objective criterion to form homogeneous groups that maximize the explained variance (R²) of all endogenous latent variables in the PLS path model. Using a deterministic approach for the assignment of observations to groups, it takes the entire path model’s structure into account and reassigns observations only if reassigning observations improves the objective criterion and thereby has no distributional assumptions [23].

### Measures and selection of indicator variables

No consensus exists on the number of indicator variables to include in a latent class analysis model [24]; however, the selection of variables should be based on a theoretical rationale that makes interpreting the model and identifying the classes valid. With our primary objective of defining subgroups that would enable the determination of key drivers of the acceptability of vaginal dosage forms, we constructed the FIMIX-PLS model using only variables related to treatment acceptability domains. These variables were used to measure a latent index of acceptability to vaginal dosage forms as part of the GSM-SVTAQ questionnaire, described elsewhere [14]. In our latent segment model, we focused on acceptability grouping rather than grouping with covariates. We included four indicator variables covering the domains of the TFA [25].

### Class solution selection

We started with a one-class model and then specified models with an additional class at a time until the best model was identified. The final selected model was based on both statistical criteria and interpretability based on our theoretical understanding of treatment acceptability. Statistical criteria included the Bayesian information criterion (BIC) as the most reliable fit statistic [17,26]. In addition, we examined the Akaike information criterion (AIC), the Sample-size Adjusted Bayesian Information Criterion (SABIC), and the Consistent Akaike Information Criterion (CAIC). A lower BIC and information criterion (ICs) are indicative of a better fit. Sarstedt et al. (2011a) demonstrated that AIC3 and CAIC can be considered jointly if both criteria indicate the same number of segments. To ensure the interpretability of the selected model, an entropy-based measure of 0.7 or higher was considered. We also considered the sizes of segments generated with no segment not to have less than 5% of the total cases to ensure that the model made conceptual sense [27].

### Sample size estimation

Determining the sample size for the FIMIX-PLS model presents a challenge due to the method’s complexity and lack of concrete guidelines. There is no fixed minimum sample size for a latent class analysis as it depends on the condition studied, the quality of indicator variables included in the model and how well the classes are separated. However, drawing from previous methodological studies, a sample size in the range of 300–1000 is acceptable, given that fit indices of the model are expected to function adequately [28–30]. While power analysis methods are not directly applicable to FIMIX-PLS, we aimed to ensure sufficient statistical power for our analyses. Considering an anticipated medium effect size (f² = 0.2), a significance level of 0.05, and a desired power of 0.8, a minimum sample size of 342 was required to detect meaningful differences between the latent classes, given that our model comprised four latent variables and six indicator variables.

### Data structuring

Indicators with missing values were case-wise deleted from the data. For our data, there were three cases with missing values, and we excluded them from the final analysis. The case-wise deletion was chosen because the mean value replacement is not recommended for use in FIMIX-PLS. It may generate artificial segments [19]. All variables were measured on the same 5-point Likert scale, eliminating the need for standardization. To test for local dependency within a class to avoid bias and lower model accuracy [31], we examined the residual correlations between indicator variables and eliminated any pair that showed correlation coefficients greater than 0.5 [32].

## Results

### Descriptives

Table 1. presents the sample demographic characteristics. A total of 351 participants were included in the final model. The mean age was 46.91 years, and the standard deviation was 6.46 years.

**Table 1:** Participant Demographic Characteristics.

Variable	Categories	Frequency per Category (%)
**Partner**	No	113 (32%)
	Yes	
**Education**		
	Graduated from College	181(51.57%)
	Completed Graduate School	88 (20.07%)
	Graduate from High School	80 (22.79%)
	Did not Attend Primary School	2 (0.57)
**Menopause**	No	187 (53%)
	Yes	164 (47%)
**Use of Vaginal Treatments**	No	180 (51%)
	Yes	171 (49%)
**Previous Use of Vaginal Treatments**	No	259 (74%)
	Yes	92 (26%)

### Identification of the latent segments

The results of FIMIX-PLS supported our hypothesis that different unobserved segments of acceptability existed among our sample. Table 2. presents segments of different class models. As shown in Table 2, the BIC and CAIC. suggested a two-segment model. Additionally, the two-segment model showed an adequate entropy of > 0.9. Because the smallest segment in the three-segment model decreased to below 5%, we selected the two-segment model.

**Table 2:** Model Fit and Diagnostic Criteria.

	Model Fit Criteria						
	AIC	AIC3	AIC4	BIC	CAIC	HQ	MDL5
**Segment 1**	2631.189	2639.189	2647.189	2662.076	2670.076	2643.482	2849.621
**Segment 2**	1894.477	1911.477	1928.477	**1960.11**	**1977.11**	1920.599	2358.644
**Segment 3**	1875.618	1901.618	1927.618	1975.999	2001.999	1915.569	2585.521
**Segment 4**	1827.514	1862.514	1897.514	1962.641	1997.641	1881.294	2783.151
	**Diagnostic Criteria**						
	**LnL**	**EN**	**NFI**	**NEC**	**Smallest Class Size (%)**		
**Segment 1**	−1307.595	0	0	0	100		
**Segment 2**	−930.238	**0.931**	0.961	24.05	44.4		
**Segment 3**	−911.809	0.858	0.888	49.91	4.7		
**Segment 4**	−878.757	0.844	0.839	54.861	6.9		

Note: The model became unstable with the five-segment model. The bold text indicates the model fit criteria. AIC = Akaike’s information criterion, BIC = Bayesian information criterion, CIAC = Consistent AIC, HQ = Hannan–Quinn criterion, MDL5 = minimum description length with factor 5, LnL = LogLikelihood, EN = normed entropy statistic, NFI = nonfuzzy index, NFC = normalized entropy criterion.

### Validation of the segmentation

The measurement model supported the reliability and validity of the measures [20,33,34]. For both segments, all indicator variables showed acceptable composite reliability (*ρ*_A_) and average variance extracted values. For discriminant validity, all variables showed a heterotrait-monotrait below 0.9. The construct reliability and discriminant validity of the indicator variables are shown in Table 3.

**Table 3:** Construct Reliability and Discriminant Validity of Indicator Variables.

Construct Reliability				
	Cronbach’s alpha	(*ρ*_A_)	Average Variance Extracted	
**Segment 1**				
Intervention Coherence	0.775	0.808	0.814	
Perceived Effectiveness	0.691	0.691	0.764	
**Segment 2**				
Intervention Coherence	0.794	0.794	0.829	
Perceived Effectiveness	0.779	0.802	0.818	
**Discriminant Validity**				
	**Affective Attitude**	**Intervention Coherence**	**Perceived Effectiveness**	**Self-Efficacy**
**Segment 1**				
Affective Attitude				
Intervention Coherence	0.735			
Perceived Effectiveness	0.889	0.456		
Self-Efficacy	0.461	0.594	0.557	
**Segment 2**				
Affective Attitude				
Intervention Coherence	0.598			
Perceived Effectiveness	0.081	0.795		
Self-Efficacy	0.527	0.701	0.506	

### The Partial Least Squares-Prediction Oriented Segmentation (PLS-POS)

The PLS-POS was run with two segments, resulting in substantial segments with sufficient representations. There were 179 (50.9%) patients in the first segment and 172 (49.1%) in the second segment. Table 4 shows the R^2^ values of the original sample and the weighted average R^2^ of each endogenous latent variable. All weighted average R^2^ values were greater than those of the original sample, providing support for heterogeneity. A higher R^2^ indicated that segmentation increased the predictive in-sample power compared to aggregate-level analysis.

**Table 4:** PLS-POS Results.

	R^2^	Weighted Average R^2^	Segment 1	Segment 2
Intervention Coherence	0.333	0.356	0.427	0.283
Perceived Effectiveness	0.229	0.572	0.623	0.519
Self-Efficacy	0.328	0.332	0.276	0.390

### Bootstrapping multigroup analysis

Before we performed the PLS bootstrapping, we ran a multi-group analysis to examine whether the resulting differences between the segments from the PLS-POS were significant. As shown in Table 5, since none of the confidence intervals (bias-corrected) of all the paths overlapped, all the paths were considered significant.

**Table 5:** Interval Confidence (Bisa Corrected) of Paths of Segments 1 & 2.

	Segment 1		Segment 2	
	2.50%	97.50%	2.50%	97.50%
Affective Attitude - > Intervention Coherence	0.542	0.739	0.351	0.678
Affective Attitude - > Perceived Effectiveness	0.721	0.96	−0.565	−0.295
Intervention Coherence - > Perceived Effectiveness	−0.491	−0.182	0.548	0.865
Intervention Coherence - > Self Efficacy	0.336	0.67	0.401	0.788
Self-Efficacy - > Perceived Effectiveness	0.123	0.362	0.057	0.391

### Partial least squares-bootstrapping

Comparing the parameters of the two segments revealed significant differences in the structural model as shown in Table 6.

**Table 6:** Partial Least Squares Bootstrapping Results.

	Original Sample	Segment 1	Segment 2
**Affective Attitude - > Intervention Coherence**	0.577***	0.654***	0.532***
**Affective Attitude - > Perceived Effectiveness**	0.174**	0.845***	−0.424***
**Intervention Coherence - > Perceived Effectiveness**	0.201**	−0.336***	0.707***
**Intervention Coherence - > Self-Efficacy**	0.573***	0.526***	0.625***
**Self-Efficacy - > Perceived Effectiveness**	0.198**	0.248***	0.235**

*** p ≤ 0.01; **p ≤ 0.05.

### Entire sample

For the entire sample, all hypothesized direct relationships were positive and statistically significant. A positive Affective Attitude significantly influenced Intervention Coherence (β = .577, p < 0.001), Perceived Effectiveness (β = .174, p < 0.01), and Self-Efficacy (β = .573,p < .001). Similarly, Intervention Coherence positively predicted both Perceived Effectiveness (β = .201, p < 0.01) and Self-Efficacy (β = .573, p < 0.001). Finally, Self-Efficacy demonstrated a significant positive effect on Perceived Effectiveness (β = .198, p < .01).

### Segment 1

A notably strong positive path was observed from Affective Attitude to Perceived Effectiveness (β = .845, p < 0.001), indicating that for individuals in this segment, positive emotional responses are a primary driver of their belief in the intervention’s effectiveness. While Affective Attitude also strongly influenced Intervention Coherence (β = .654, p < 0.001) and Intervention Coherence positively predicted Self-Efficacy (β = .526, p < 0.001), the direct relationship between Intervention Coherence and Perceived Effectiveness was negative (β=−.336, p < .001). Self-Efficacy continued to positively influence Perceived Effectiveness (β = .248, p < 0.001).

### Segment 2

Intervention Coherence emerged as the strongest direct predictor of Perceived Effectiveness (β = .707, p < 0.001), highlighting the critical role of cognitive understanding in shaping their belief in the intervention’s efficacy. Interestingly, the direct path from Affective Attitude to Perceived Effectiveness was significantly negative (β=−.424, p < 0.001), suggesting that positive feelings alone do not directly lead to Perceived Effectiveness in this group, and may even have a counter-intuitive direct effect when other factors are considered. Other relationships remained robust: Affective Attitude positively influenced Intervention Coherence (β = .532, p < 0.001), and Intervention Coherence strongly predicted Self-Efficacy (β = .625, p < 0.001). Self-Efficacy also maintained a positive influence on Perceived Effectiveness (β = .235, p < 0.01).

### Model explanation

**Fig 3:**
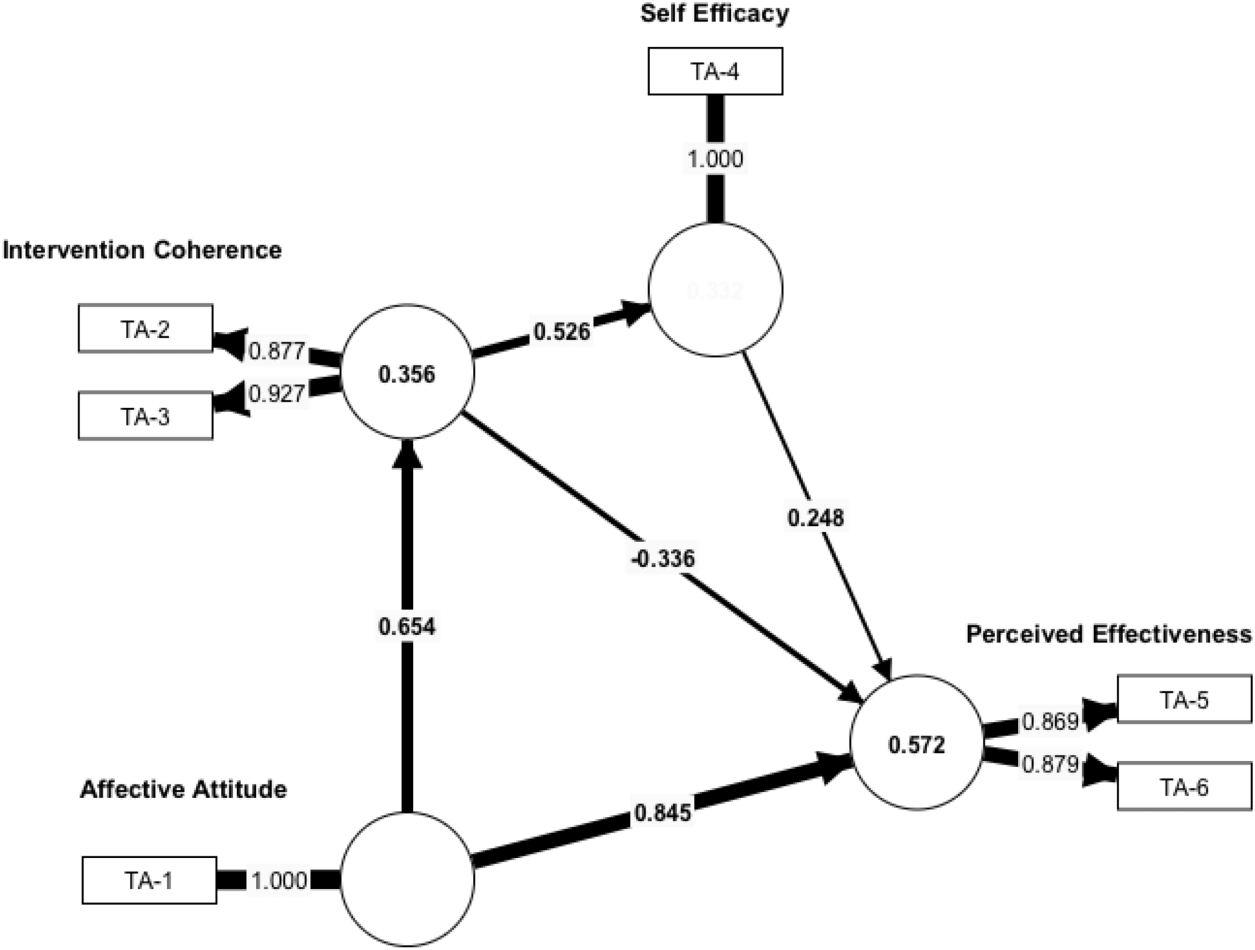
Path Coefficients and Outer Loadings for Segment 1.

**Fig 4:**
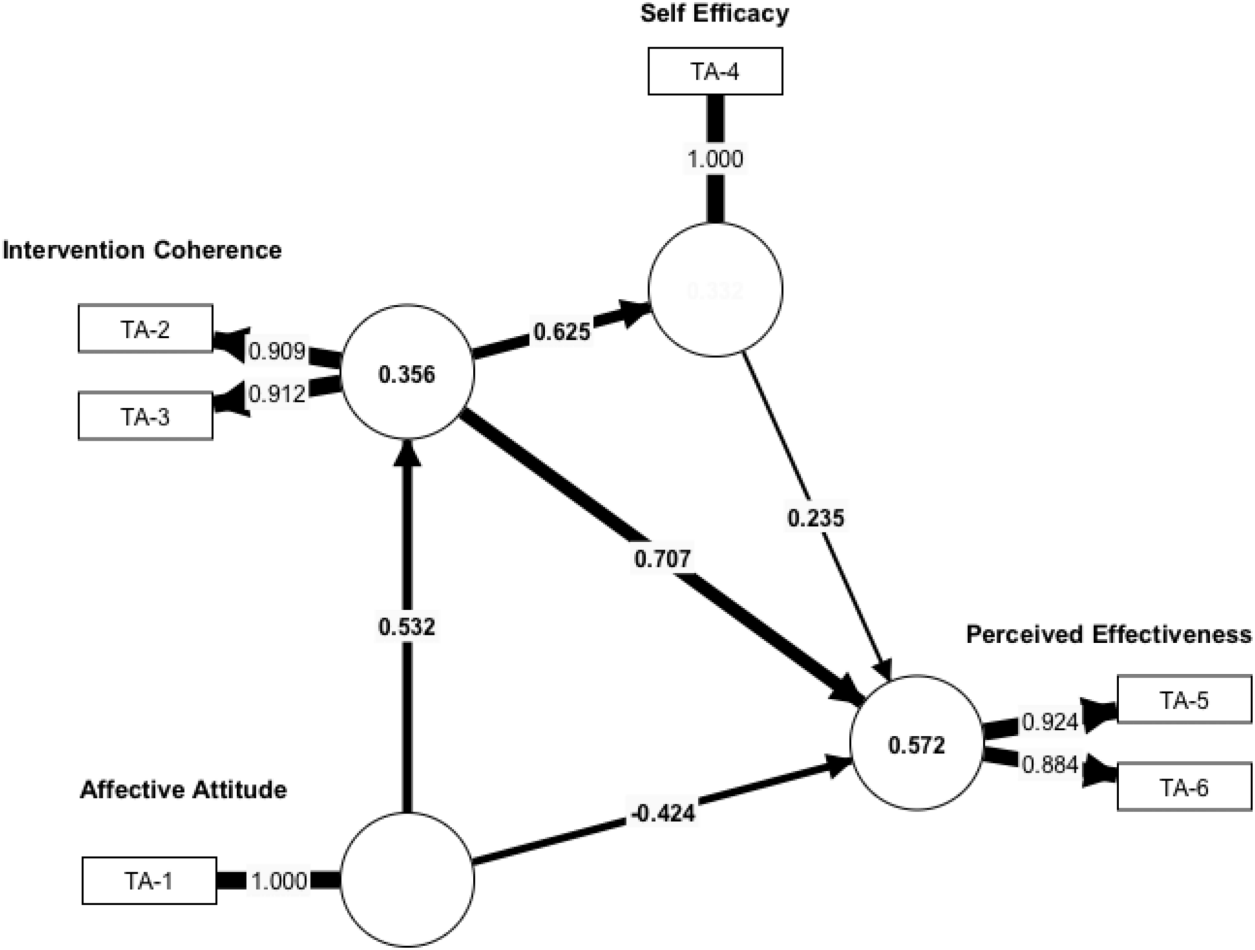
Path Coefficients and Outer Loadings for Segment 2.

Comparing the cell counts, we found that the best match was achieved when respondents who were not receiving vaginal treatment were assigned to POS segment 1 (*n* = 180) and those who were receiving vaginal treatment were assigned to POS segment 2 (*n* = 171). Affective Attitude was the most important predictor of Perceived Effectiveness for those who were not receiving vaginal treatment (Seg1-NOT), whereas for those who were receiving vaginal treatment (Seg1-ARE), Intervention Coherence was the most important predictor. Self-efficacy was significantly correlated with Perceived Effectiveness in both patient segments. Fig 3 and Fig 4 show the path coefficients and outer loadings for segment 1 and 2, respectively.

## Discussion

Our analysis identified two latent segments of treatment acceptability for vaginal dosage forms among peri- and postmenopausal women. The first segment was observed among women who never used a vaginal dosage form for symptom management. Patients in this group were mainly concerned about the overall expected comfort level with the use of vaginal treatments. The second segment was observed among women who were currently using or had recent experience using vaginal treatments. Patients in this group were mainly concerned about the extent to which they understood their treatment and how it worked. This confirms what Sekhon and colleagues have proposed in their development of the TFA that the measurement of intervention acceptability should distinguish between anticipated and experienced acceptability [25].

The model showed a significant positive correlation between intervention coherence and perceived effectiveness among patients with previous use of vaginal treatments. It was suggested that a patient’s previous experiences and beliefs about treatment can be an influential factor in the patient’s decision-making, independent of her received information about the effectiveness of the treatments [35]. These results emphasize that familiarity with a specific route of delivery is an important determinant of acceptability and may influence women’s preferences for treatments [5]. Due to their versatility in terms of dosage and frequency of administration, low-dose estrogen creams are the most widely used vaginal products for the treatment of GSM. Long-acting estradiol-releasing rings are also preferable and require less sustained effort to apply [36]. We may conclude that patients with previous treatment experiences will tend to accept treatment more than those who are naïve to it, or that the perception of acceptability could change with the actual experience of the treatment.

On the other hand, how a patient feels about her treatment and her anticipated level of comfort or burden are driven by her choice of treatment. In our study, we concluded that for women who were not engaged in vaginal treatments, their beliefs were driven by their affective attitude toward the treatment. We emphasize that patients’ involvement in treatment decision-making could greatly improve their acceptance of using a vaginal treatment. Acceptability may be influenced by vaginal practices that differ cross-culturally and involve a level of comfort with touching the genital areas [5]. This was previously observed by Gigurer and colleagues in a study that compared American women with Puerto Rican women in their acceptability to use a vaginal gel. American women reported negative associations, whereas Puerto Rican women experienced them in a positive manner [37].

Assessment of acceptability from a temporal perspective (pre- vs post-intervention) could be a factor that influences a patient’s willingness to try a vaginal dosage form. It has been proposed that the perception of acceptability is a dynamic concept that could change with the experience of the treatment [38]. Acceptability of treatment is an integral part of the success of any effective treatment. Patients who are more accepting of treatment tend to adhere more to it. Adherence and acceptability are two different but potentially related [39–40]. Adherence explains a patient’s behaviour toward treatment, but acceptance predicts that behaviour [35]. In turn, there is a relationship between a patient’s perception of self-efficacy and adherence to treatment. Considering this complex multifactorial relationship, our model showed a significant positive correlation between patients’ confidence in how they can engage in behaviours required to adhere to treatment and their perception of effectiveness in both groups.

The study’s generalizability is limited by its cross-sectional design and reliance on patient-reported data, which may introduce respondent and recall bias. Cultural and background differences can have a significant impact on how people perceive different aspects of their treatments and, therefore caution is warranted when attempting to extrapolate the study’s conclusions to populations that may possess distinct cultural or demographic characteristics. Additionally, the use of purposive sampling, may further limit the generalizability of the findings to the broader population with distinct characteristics.

### Study implications

Many important gaps in research on the acceptability of vaginal treatments among perimenopausal and postmenopausal women with GSM remain unaddressed. Many social and cultural factors have contributed to these gaps being neglected and not an area of research focus. Despite the many studies that examined different aspects of the acceptability of vaginal dosage forms, studies among menopausal women have not yet received much attention. The validated FIMIX-PLS model provided the optimal approach to understanding unobserved heterogeneity between users of vaginal treatments. The model identified differences in acceptability between current/former and naïve users. Identification of different segments of perceived effectiveness within this population and the factors that predict and mediate them emphasizes the importance of the patient’s informed choice of vaginal treatment. It also emphasizes the importance of understanding the complex relationships between these predictors and mediators that shape menopausal women’s perception and acceptance of their treatments. This shall inform healthcare policies and clinical guidelines on the importance of incorporating factors influencing the acceptability of vaginal treatments among menopausal women. The model serves as a valuable benchmark and reference for healthcare professionals, especially pharmacists, advocating for a more patient-centred perspective and emphasizing the importance of sociocultural variations in women’s perceptions of vaginal treatments. On the patient level, the findings raise awareness about the challenges faced by menopausal women regarding their treatment options. Addressing the acceptability of vaginal treatments shall help reduce the stigma and burden and empower patients in their treatment decisions.

## Conclusion

For many urogenital conditions, vaginal drug delivery remains one of the most efficient routes, with many advantages over other routes of administration. Unlike other routes that require negligible effort, the application of, adherence to, and satisfaction with vaginal treatment can cause cognitive and physical distress to the patient. Therefore, a woman’s acceptance of vaginal treatment cannot be overlooked. It is important to understand the aspects related to and factors influencing a woman’s willingness to use vaginal treatments. Clinical guidelines remain silent on most of these aspects. A great research area of focus should be directed toward exploring sociocultural variations in beliefs, perceptions, and barriers to using vaginal treatments in an attempt to enhance women’s acceptability.
